# Association of Leisure-Time Physical Activity Across the Adult Life Course With All-Cause and Cause-Specific Mortality

**DOI:** 10.1001/jamanetworkopen.2019.0355

**Published:** 2019-03-08

**Authors:** Pedro F. Saint-Maurice, Diarmuid Coughlan, Scott P. Kelly, Sarah K. Keadle, Michael B. Cook, Susan A. Carlson, Janet E. Fulton, Charles E. Matthews

**Affiliations:** 1 Division of Cancer Epidemiology and Genetics, National Cancer Institute, Rockville, Maryland; 2 Division of Cancer Control and Population Sciences, National Cancer Institute, Rockville, Maryland; 3 Health Economics and Evidence Synthesis Group, Institute of Health and Society, Newcastle University, United Kingdom; 4 Department of Kinesiology and Public Health, California Polytechnic State University, San Luis Obispo, California; 5 Division of Nutrition, Physical Activity, and Obesity, National Center for Chronic, Disease Prevention and Health Promotion, Centers for Disease Control and Prevention, Atlanta, Georgia

## Abstract

**Importance:**

Although the benefits of leisure-time physical activity (LTPA) in middle age are established, the health effects of long-term participation and changes in LTPA between adolescence and middle age have not been documented.

**Objective:**

To determine whether an association exists between LTPA life course patterns and mortality.

**Design, Setting, and Participants:**

This prospective cohort study used data from the National Institutes of Health–AARP (formerly American Association of Retired Persons) Diet and Health Study established in 1995 to 1996. Data analysis was conducted from March 2017 through February 2018. Data were analyzed for 315 059 adult AARP members living in 6 states, namely, California, Florida, Louisiana, New Jersey, North Carolina, or Pennsylvania, or 2 metropolitan areas, Atlanta, Georgia, or Detroit, Michigan.

**Exposures:**

Self-reported LTPA (hours per week) at the baseline interview for ages grouped as 15 to 18, 19 to 29, 35 to 39, and 40 to 61 years.

**Main Outcomes and Measures:**

All-cause, cardiovascular disease (CVD)–related, and cancer-related mortality records available through December 31, 2011.

**Results:**

Of 315 059 participants, 183 451 (58.2%) were men, and the participants were 50 to 71 years of age at enrollment. Ten LTPA trajectories (categorized as maintaining, increasing, and decreasing LTPA across time) were identified, and 71 377 deaths due to all causes, 22 219 deaths due to CVD, and 16 388 deaths due to cancer occurred. Compared with participants who were consistently inactive throughout adulthood, participants who maintained the highest amount of LTPA in each age period were at lower risks for all-cause, CVD-related, and cancer-related mortality. For example, compared with participants who were consistently inactive, maintaining higher amounts of LTPA was associated with lower all-cause (hazard ratio [HR], 0.64; 95% CI, 0.60-0.68), CVD-related (HR, 0.58; 95% CI, 0.53-0.64), and cancer-related (HR, 0.86; 95% CI, 0.77-0.97) mortality. Adults who were less active throughout most of the adult life course but increased LTPA in later adulthood (40-61 years of age) also had lower risk for all-cause (HR, 0.65; 95% CI, 0.62-0.68), CVD-related (HR, 0.57; 95% CI, 0.53-0.61), and cancer-related (HR, 0.84; 95% CI, 0.77-0.92) mortality.

**Conclusions and Relevance:**

Maintaining higher LTPA levels and increasing LTPA in later adulthood were associated with comparable low risk of mortality, suggesting that midlife is not too late to start physical activity. Inactive adults may be encouraged to be more active, whereas young adults who are already active may strive to maintain their activity level as they get older.

## Key Points

**Question:**

Does an association exist between patterns in leisure-time physical activity occurring during adolescence (15-18 years of age) or early (19-29 years of age), middle (35-39 years of age), and later (40-61 years of age) adulthood and all-cause or cause-specific mortality?

**Findings:**

This cohort study of 315 059 participants found that maintaining physical activity from adolescence into later adulthood was associated with 29% to 36% lower risk for all-cause mortality and that being inactive but increasing physical activity during midlife was associated with 32% to 35% lower risk for mortality.

**Meaning:**

Although long-term participation in physical activity may be important to lower mortality risk, the present study provides evidence that becoming physically active later in adulthood (40-61 years of age) may provide comparable health benefits.

## Teaser

This cohort study analyzes questionnaire data from the National Institutes of Health–AARP (formerly American Association of Retired Persons) Diet and Health Study to assess whether an association exists between risk of all-cause, cardiovascular disease–related, or cancer-related mortality and self-reported leisure-time physical activity patterns across the life course.

## Introduction

National guidelines for aerobic physical activity recommend adults should participate in at least 150 minutes per week of moderate intensity aerobic activity or 75 minutes per week of vigorous intensity activity or an equivalent combination of both.^1,2,3^ Levels of physical activity equivalent to meeting this guideline have been associated with substantial health benefits, including reductions in all-cause,^4,5^ cardiovascular disease (CVD)–related,^4,6^ and cancer-related^4,7,8^ mortality. However, most of the evidence on the mortality benefits of physical activity comes from studies that measure leisure-time physical activity (LTPA) at only 1 point, usually during midlife (eg, 40-60 years of age). To date, no study to our knowledge has taken a life course approach to examine how participation in physical activity throughout the different stages of adulthood may be associated with mortality risk.

Little is known about how long-term participation in LTPA from adolescence to early adulthood and into middle age may affect mortality. There have been studies conducted on mortality risk from maintaining or changing LTPA during adulthood,^9,10,11,12,13,14^ although those studies primarily examined changes in physical activity occurring during midlife for short periods of time (eg, 2-7 years), but not from adolescence into middle and older ages. Furthermore, only 2 of the midlife change studies examined cause-specific mortality. Thus, it is unclear whether there are differential associations between LTPA life course patterns and death due to CVD or cancer.^11,15^ Understanding the optimal patterns of LTPA (eg, maintained, increased, or decreased) in relation to all-cause and cause-specific mortality is particularly important because this evidence can help identify health-enhancing patterns of physical activity throughout the adult life course. Accordingly, the goal of this study was to examine how patterns in LTPA occurring over a wide age range, that is, adolescence (15-18 years) and early (19-29 years), middle (35-39 years), and later adulthood (40-61 years), are associated with all-cause and cause-specific (ie, CVD and cancer) mortality. We hypothesized that participants who maintained the highest levels of activity in all age groups would have the lowest risk for mortality.

## Methods

### Study Design

We used data collected in the National Institutes of Health (NIH)–AARP (formerly American Association of Retired Persons) Diet and Health Study (1995-1996), and our data analysis was conducted from March 2017 through February 2018. This NIH-AARP study included a baseline and a Risk Factor Questionnaire^16^ that was initially administered from 1995 to 1996 to AARP members 50 to 71 years of age living in 6 states, namely, California, Florida, Louisiana, New Jersey, North Carolina, or Pennsylvania, or living in 2 metropolitan areas, Atlanta, Georgia, or Detroit, Michigan. Information collected from these 2 questionnaires was merged with National Death Index mortality records available through December 31, 2011. The underlying cause of death was determined using the *International Classification of Diseases, Ninth Revision* (*ICD-9*) and classified as all-cause, CVD (*ICD-9* codes: 390-398, 401-404, 410-438, 440-448), or cancer. This study follows the guidelines for reporting cohort studies using the Strengthening the Reporting of Observational Studies in Epidemiology (STROBE) reporting guideline.^17^ The NIH-AARP Diet and Health Study was reviewed and approved by the Special Studies Institutional Review Board of the US National Cancer Institute, and all participants gave written informed consent by completing and returning the questionnaire.

### Analytic Sample

A baseline questionnaire was initially sent to 3.5 million participants and was returned by 566 398 participants who had adequately completed it. Soon after, 539 213 participants eligible for the study (ie, not reporting as having colorectal cancer, breast cancer, prostate cancer, or renal disease) were mailed a second questionnaire (the Risk Factor Questionnaire), and 334 905 participants returned this survey. We excluded participants whose questionnaires were completed by proxies (n = 10 383) or had missing LTPA item scores (n = 9463). Because the earliest exposure was late adolescence (15-18 years), a life period that is likely to precede the onset of most comorbid conditions, we made no further exclusions for health indicators reported at baseline. The final analytic sample included 315 059 records.

### Leisure-Time Physical Activity

The Risk Factor Questionnaire included a question about previous participation in moderate to vigorous physical activity at 15 to 18 years of age, 19 to 29 years of age, and 35 to 39 years of age and the previous 10 years, estimated to be in the range of 40 to 61 years based on age at study baseline (eFigure 1 in the Supplement). We examined the 6-month test-retest reliability of this item in a parallel study of 870 AARP members (50-74 years old) enrolled in the Interactive Diet and Activity Tracking in AARP.^18^ We found intraclass correlations of 0.55 for physical activity at 15 to 18 years of age, 0.53 for physical activity at 19 to 29 years of age, 0.52 for physical activity at 35 to 39 years of age, and 0.55 for physical activity in the previous 10 years. Information ascertained from this item was coded as follows: 0 hours per week (rarely or never), 0.5 (weekly but <1 hour), 2.0 (1-3 hours/wk), 5.5 (4-7 hours/wk), and 9 (>7 hours/wk) of LTPA at each life period.

### Statistical Analysis

#### Life Course LTPA Trajectories

Using semiparametric group-based mixture models, we first modeled time spent in LTPA at each life period to identify LTPA trajectories.^19,20^ We determined the number of trajectories using the Bayesian information criterion associated with the models and the sample sizes in the final groups. For each trajectory, we described the membership probability (ie, likelihood that a participant’s individual life course LTPA pattern matched that of a certain trajectory) determined using maximum likelihood estimators with polynomial functions of LTPA reported at each time (ie, age groups). With this approach, participants were assigned to trajectories that more closely matched their observed values or trajectory that resulted in the highest membership probability, which in most models resulted in probabilities of 0.9 or greater. High membership probabilities assure homogeneity and support exclusiveness of trajectory assignment.

#### Associations With All-Cause, CVD-Related, and Cancer-Related Mortality

We examined the associations between each trajectory group (representing a life-course LTPA pattern) with all-cause, CVD-related, and cancer-related mortality end points using trajectory 1, which was consistently inactive (rarely or never engaged in LTPA at age groups of 15-18, 19-29, 35-39, and 40-61 years) as the referent group. Mortality outcomes using follow-up time (in years; starting follow-up at Risk Factor Questionnaire) were modeled using Cox proportional hazards models with a set of covariates and trajectory group as independent variables. The analysis focused on fully adjusted models, but we also examined LTPA-mortality associations using reduced models to understand the contributions of different covariates to our risk estimates. Covariates (assessed in the baseline questionnaire) included age, sex, race/ethnicity, educational level, smoking status/dose, body mass index (BMI, calculated as weight in kilograms divided by height in meters squared) category at 18 years of age, total energy intake, diet percent fat, red meat consumption, alcohol consumption, vegetable consumption, fruit consumption, and vitamin/mineral supplementation.

#### Sensitivity Analyses

We conducted a sensitivity analysis of the hazard ratios (HRs) after excluding early death (first 2 years) and also adjusting or excluding for disease conditions at baseline to examine for reverse causality. Given that there was some noticeable variability within trajectories, we also conducted a sensitivity analysis limited to participants with more homogeneous distributions of LTPA scores (ie, participants with probabilities of trajectory assignment lower than 0.80 were excluded). Additional examinations included fully adjusted Cox models stratified by sex and BMI at 50 to 71 years of age (ie, baseline) to examine whether associations with all-cause mortality were similar between men and women, and across BMI classifications. Analyses related to BMI adjusted for BMI at 18 years of age and were stratified by BMI at baseline to understand whether changes in BMI as a result of increased or decreased participation in LTPA during the adult life course were contributing to some of the associations between LTPA and mortality outcomes.

## Results

### Life Course LTPA Trajectories

Of the 315 059 participants, 183 451 (58.2%) were male, and participants were 50 to 71 years of age at enrollment. There were 71 377 deaths from all causes, 22 219 from CVD, and 16 388 from cancer during a mean (SD) follow-up of 13.6 (3.3) years. We identified 10 trajectories of LTPA that were labeled based on the last end point for LTPA (40-61 years of age) relative to the first LTPA end point (15-18 years of age). We classified trajectories into 3 categories: participants with consistently higher or stable LTPA over time (maintainers; 176 654 or 56.1% of the total sample; including the referent group); participants indicating an increase in LTPA from adolescence or later in adulthood (increasers; 41 193 or 13.1%); or participants with patterns of higher early adulthood LTPA but reduced activity later in adulthood (decreasers; 97 212 or 30.8%) (Table). For example, participants in trajectory 10 accumulated similar amounts of LTPA when they were 15 to 18 years old and when they were 40 to 61 years, and in the middle years maintained some level of activity; hence, they were labeled maintainers. Additional detailed information about the distributions is available in eTable 1 and eTable 2 in the Supplement.

**Table.** Descriptive Characteristics by LTPA Trajectory, National Institutes of Health–AARP Diet and Health Study From 1995 to 2011

Characteristic	LTPA Trajectories^a^									
	Maintainers					Increasers		Decreasers		
	1 [Reference]	5	7	8	10	2	3	4	6	9
No. (%)	13 170 (4.2)	48 195 (15.3)	72 783 (23.1)	32 608 (10.4)	9898 (3.1)	25 094 (8.0)	16 099 (5.1)	16 611 (5.3)	35 846 (11.4)	44 755 (14.2)
Age, mean (SD), y	63.0 (5.3)	62.6 (5.4)	63.2 (5.2)	63.0 (5.3)	63.1 (5.2)	63.0 (5.4)	62.9 (5.4)	63.3 (5.3)	62.8 (5.2)	62.1 (5.4)
Male, No. (%)	6588 (50.0)	24 963 (51.8)	42 868 (58.9)	21 875 (67.1)	7347 (74.2)	13 675 (54.5)	5677 (35.3)	7155 (43.1)	22 492 (62.8)	30 811 (68.8)
Non-Hispanic white, No. (%)	11 840 (89.9)	44 164 (91.6)	68 154 (93.6)	30 742 (94.3)	9342 (94.4)	23 390 (93.2)	15 045 (93.5)	15 499 (93.3)	33 161 (92.5)	41 312 (92.3)
Educational level <high school, No. (%)	1235 (10.2)	2671 (5.5)	3460 (4.7)	952 (2.9)	224 (2.3)	1136 (4.5)	902 (5.6)	795 (4.8)	1517 (4.3)	1538 (3.5)
Smoke >20 cigarettes/d, No. (%)	645 (4.9)	1889 (3.9)	2815 (3.9)	887 (2.7)	165 (1.7)	453 (1.8)	455 (2.8)	786 (4.7)	2137 (6.0)	2207 (4.9)
LTPA of age group, mean (SD), h/wk										
15-18 y	0.2 (0.6)	1.3 (1.1)	8.5 (1.2)	7.3 (1.8)	6.8 (1.7)	1.1 (0.9)	1.6 (1.0)	3.3 (2.0)	8.7 (1.0)	7.0 (1.7)
19-29 y	0.0 (0.1)	1.3 (1.2)	8.8 (0.9)	5.5 (1.6)	1.7 (0.9)	1.4 (1.2)	6.2 (2.2)	5.5 (1.7)	8.1 (1.5)	3.7 (2.1)
35-39 y	0.0 (0.0)	1.2 (1.2)	8.4 (1.3)	4.9 (1.8)	1.9 (1.5)	2.8 (2.2)	7.5 (1.8)	5.8 (1.8)	6.2 (2.2)	1.6 (1.1)
40-61 y	0.0 (0.0)	1.0 (0.9)	7.7 (1.7)	6.5 (1.6)	6.9 (1.7)	6.6 (1.6)	7.5 (1.7)	1.6 (0.8)	1.4 (0.8)	1.1 (0.9)
BMI, No. (%)										
At 18 y ≥30.0	357 (2.7)	942 (2.0)	780 (1.1)	358 (1.1)	102 (1.0)	464 (1.9)	247 (1.5)	275 (1.7)	552 (1.5)	736 (1.6)
Current ≥30.0	3611 (27.4)	11 052 (22.9)	12 252 (16.8)	4627 (14.2)	1285 (13.0)	3015 (12.0)	2406 (15.0)	4124 (24.8)	10 762 (30.0)	11 783 (26.3)
Calories, mean (SD), kcal/d per 1000	1.8 (1.0)	1.7 (0.9)	2.0 (1.0)	1.9 (0.8)	1.8 (0.8)	1.7 (0.8)	1.7 (0.9)	1.7 (0.8)	1.9 (1.0)	1.9 (0.9)
Dietary fat, mean (SD), % kcal from fat	31.3 (8.0)	30.4 (7.7)	29.9 (7.7)	29.2 (7.6)	28.0 (7.5)	28.0 (7.7)	29.2 (7.7)	30.8 (7.6)	31.1 (7.6)	30.8 (7.6)
Red meat, mean (SD), g/d	65.3 (76.4)	60.9 (59.1)	68.7 (63.5)	62.9 (57.9)	56.3 (49.3)	51.0 (49.1)	54.2 (51.9)	61.5 (55.2)	75.5 (66.8)	71.7 (61.3)
Alcohol, mean (SD), g/d	11.7 (41.0)	11.3 (33.7)	14.0 (36.8)	13.8 (32.5)	14.7 (33.3)	12.2 (31.4)	11.0 (30.9)	11.1 (32.8)	14.6 (41.6)	14.1 (38.8)
Vegetables, mean (SD), portions/d	3.3 (2.5)	3.5 (2.4)	4.5 (2.9)	4.1 (2.5)	4.0 (2.4)	3.9 (2.5)	4.0 (2.6)	3.7 (2.3)	4.0 (2.7)	3.8 (2.4)
Fruit, mean (SD), portions/d	2.5 (2.4)	2.7 (2.3)	3.3 (2.7)	3.2 (2.4)	3.2 (2.4)	3.2 (2.4)	3.1 (2.6)	2.7 (2.3)	2.9 (2.4)	2.7 (2.3)
No vitamin/mineral supplementation, No. (%)	4404 (33.4)	13 783 (28.6)	19 781 (27.2)	8448 (25.9)	2465 (24.9)	5785 (23.1)	3847 (23.9)	4416 (26.6)	10 538 (29.4)	13 366 (29.9)

Abbreviations: AARP, formerly American Association of Retired Persons; BMI, body mass index (calculated as weight in kilograms divided by height in meters squared); LTPA, leisure-time physical activity.

^a^ Ten LTPA trajectories were classified into 3 categories: participants with consistently higher or stable LTPA across time (maintainers; including the referent group), those indicating an increase in LTPA from adolescence or later in adulthood (increasers), or those with patterns of higher early adulthood LTPA but reduced activity later in adulthood (decreasers).

### All-Cause and CVD-Related Mortality

**Figure 1.**
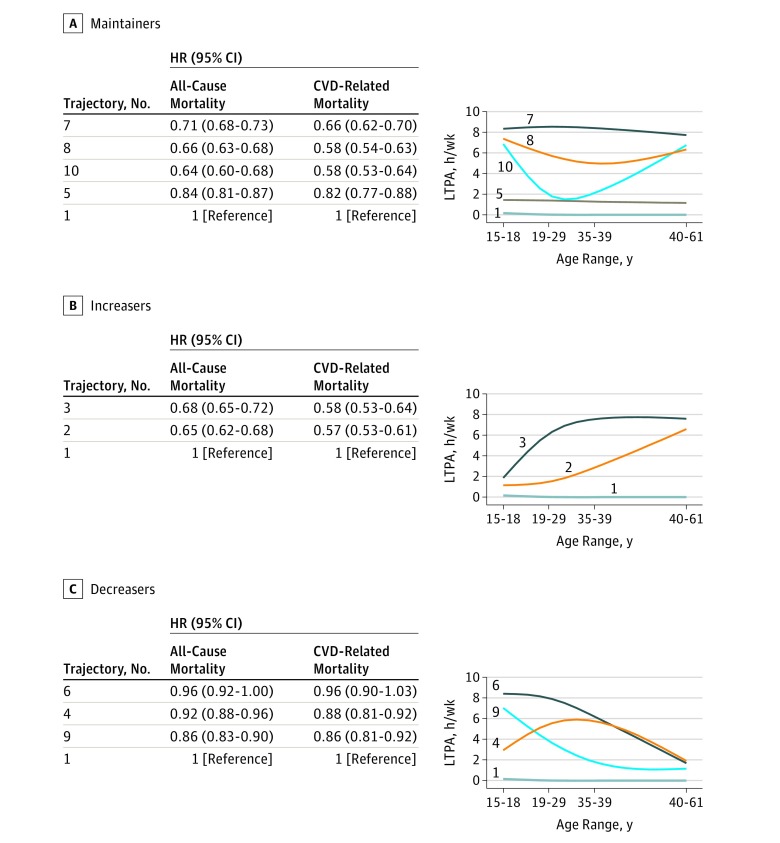
Leisure-Time Physical Activity (LTPA) Trajectories and Respective Hazard Ratios (HRs) for All-Cause and Cardiovascular Disease (CVD)–Related Mortality Among Maintainers, Increasers, and Decreasers The HRs were computed separately for all-cause and CVD-related mortality outcomes, and each model used trajectory 1 as the referent group. The HRs were adjusted for age, sex, educational level, race, smoking status/dose, total energy intake, diet percent fat, alcohol consumption, red meat consumption, fruit consumption, vegetable consumption, vitamin/mineral supplementation, and body mass index at 18 years of age.

The patterns of associations between LTPA trajectories and both all-cause and CVD-related mortality were similar. Adults who reported maintaining higher levels of LTPA in all age groups had significantly lower all-cause and CVD-related mortality compared with those who were consistently inactive over time (trajectory 1; referent group). Maintaining moderate to high amounts of LTPA (2-8 hours/wk) resulted in 29% to 36% lower risk (HRs [95% CIs]: trajectory 7, 0.71 [0.68-0.73]; trajectory 8, 0.66 [0.63-0.68]; trajectory 10, 0.64 [0.60-0.68]) compared with those consistently inactive, whereas maintaining much lower levels of LTPA (1 hour/wk; trajectory 5) was associated with only a 16% lower risk (HR, 0.84; 95% CI, 0.81-0.87). The associations for CVD-related mortality followed the same pattern as for all-cause mortality, and when compared with the consistently inactive referent group (trajectory 1), risk was 34% to 42% lower for the most active groups (eg, trajectory 10: HR, 0.58; 95% CI, 0.53-0.64) and 18% lower for the minimally active group (trajectory 5: HR, 0.82; 95% CI, 0.77-0.88) (Figure 1A; eTable 3 in the Supplement).

Participants who increased their activity level in adulthood also had a significantly lower risk for all-cause and CVD-related mortality compared with those who were consistently inactive (trajectory 1). Increasing LTPA either early or later in adulthood was associated with 32% to 35% lower mortality (Figure 1B; eTable 4 in the Supplement), which was comparable to the association observed for those who maintained higher LTPA levels from adolescence to 40 to 61 years of age (Figure 1A; eTable 3 in the Supplement). For example, those who increased LTPA in early adulthood (19-29 years of age) had 32% lower risk for all-cause mortality when compared with the referent group (trajectory 3: HR, 0.68; 95% CI, 0.65-0.72), and those who increased LTPA only later in adulthood had 35% lower risk (trajectory 2: HR, 0.65; 95% CI, 0.62-0.68). The CVD-related mortality risk for participants who increased LTPA in later adulthood (40-61 years of age) was 43% lower when compared with the referent group (trajectory 2: HR, 0.57; 95% CI, 0.53-0.61) (Figure 1B; eTable 4 in the Supplement). Individuals who reported high LTPA early in adulthood but lower levels by 40 to 61 years of age (decreasers) appeared to have little all-cause or CVD-related mortality protection in midlife (Figure 1C; eTable 4 in the Supplement).

### Cancer-Related Mortality

**Figure 2.**
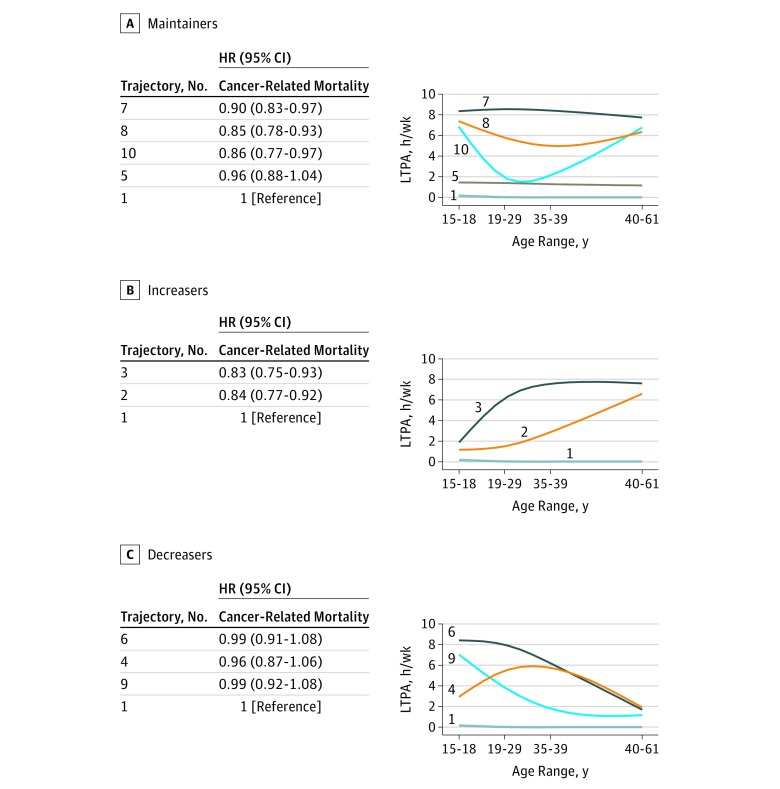
Leisure-Time Physical Activity (LTPA) Trajectories and Respective Hazard Ratios (HRs) for Cancer-Related Mortality Among Maintainers, Increasers, and Decreasers The HRs were computed separately for cancer-related mortality outcome using trajectory 1 as the referent group and were adjusted for age, sex, educational level, race, smoking status/dose, total energy intake, diet percent fat, alcohol consumption, red meat consumption, fruit consumption, vegetable consumption, vitamin/mineral supplementation, and body mass index at 18 years of age.

Maintaining moderate to high amounts of LTPA was associated with lower cancer-related mortality. For example, maintaining at least 2 to 7 hours per week resulted in 14% lower risk when compared with participants who were consistently inactive throughout adulthood (eg, trajectory 10: HR, 0.86; 95% CI, 0.77-0.97). Maintaining some LTPA (ie, 1 hour/wk throughout adult life course) was associated with similar risk for cancer-related mortality (trajectory 5: HR, 0.96; 95% CI, 0.88-1.04) (Figure 2A; eTable 3 in the Supplement).

Increasing LTPA during adulthood was associated with lower cancer-related mortality. Participants increasing LTPA in later adulthood (40-61 years of age) had a 16% lower risk when compared with the referent group (trajectory 2: HR, 0.84; 95% CI, 0.77-0.92) (Figure 2B; eTable 4 in the Supplement). There were no significant differences in risk for cancer-related mortality between participants who were consistently inactive (referent group) and those who decreased LTPA across the adult life course (Figure 2C; eTable 4 in the Supplement).

### Sensitivity Analyses

Hazard ratios remained similar when we excluded participants who died within the first 2 years of follow-up (eTable 5 and eTable 6 in the Supplement). The trends in HRs across trajectories also remained similar and were attenuated after we adjusted for or excluded prevalent health conditions (eFigure 2 in the Supplement). Excluding participants with probability assignments to a given trajectory of less than 0.80 did not alter the HRs described for our primary results (eTable 7 and eTable 8 in the Supplement). Additional examinations stratified by sex and BMI also revealed similar HRs for all-cause mortality (eFigure 3 and eFigure 4 in the Supplement) and both CVD- and cancer-related mortality although these latter 2 results are not shown.

## Discussion

This large prospective study of adults examined participation in LTPA across the adult life course and found that compared with adults consistently inactive, increasing LTPA in adulthood after being inactive during adolescence was associated with reduced risk for all-cause and cause-specific mortality. Adults who engaged in LTPA only later, by age 40 to 61 years (increasers; trajectory 2), had a risk for mortality that was comparable to those who engaged in LTPA consistently from adolescence throughout adulthood (ie, maintainers; trajectory 7). By contrast, being active in adolescence but decreasing LTPA across the adult life course was associated with smaller benefits. These findings showed that adults who became physically active later in life had mortality rates similar to those of lifelong exercisers and that most of the benefits of activity performed earlier in life (adolescence or early adulthood) were lost if activity was not maintained.

We had anticipated that participants who maintained the highest levels of activity throughout adulthood would be at lowest risk and were thus surprised to find that increasing activity early or late in adulthood was associated with comparable benefits. These benefits held similarly for men and women (eFigure 3 in the Supplement) and were independent of changes in BMI over time (eFigure 4 in the Supplement). We specifically found that being inactive across early adulthood but increasing LTPA later at 40 to 61 years (trajectory 2; increasers) was associated with 16% to 43% risk reduction in mortality. These mortality benefits were comparable to those associated with maintaining LTPA in all age groups from adolescence and into adulthood (maintainers; trajectory 7). These findings are consistent with previous studies demonstrating that increasing activity in midlife is associated with health benefits for all-cause mortality. For example, the study by Byberg et al,^12^ with the largest interval between physical activity assessments (ie, physical activity at 50 years of age and at 60 years of age), found among 1759 men followed up for 10 years, that increasing physical activity was associated with 19% to 49% lower risk for all-cause mortality. Our study extends this evidence by examining patterns of activity for as many as 46 years, enabling a more comprehensive evaluation of the timing of physical activity across the life course associated with mortality risk. To our knowledge, this is the first study to assess LTPA participation for this length of time and to examine the implications of starting or reducing LTPA at various points during adulthood. Hence, we were able to examine 10 empirically derived trajectories of LTPA reflecting long-term behavioral patterns, whereas previous studies mostly examined changes in behaviors across 2 time points (baseline and follow-up) in midlife or mean changes in activity (rate of change), limiting our understanding of the implications of increasing or decreasing physical activity in different age groups during adulthood.^10,11,12,13,15,21,22,23,24,25,26,27,28,29^

Another unique finding of the present study was that increasing or maintaining higher levels of LTPA was also associated with cause-specific mortality and the 2 leading causes of death, CVD and cancer. Increasing or maintaining LTPA was associated with 40% lower CVD-related mortality and about 15% lower cancer-related mortality when compared with the consistently inactive group. Most studies examining changes in physical activity examined only all-cause mortality, and only 2 studies examined cause-specific mortality. In 2836 Spanish adults at least 60 years of age, Higueras-Fresnillo et al^15^ found that increasing physical activity for 2 years after 60 years of age was associated with 25% lower risk of CVD-related mortality, whereas those who maintained higher activity during this period had 58% lower risk. Gregg et al^11^ assessed physical activity of 7553 women at about 71 years of age and then again 6 years later, evaluating CVD- and cancer-related mortality during 5.1 years of follow-up. Their results were mixed. Increasing physical activity was associated with 36% lower CVD and 51% cancer-related mortality, but whereas maintaining physical activity over time was associated with lower mortality risk for CVD, this was not true for cancer-related mortality. These mixed findings may be due to the older age of participants, limited statistical power for cancer-related mortality (only 264 deaths), or possibly limited assessment of physical activity levels earlier in life.^11^ Our study extends the understanding of the importance of maintaining or increasing physical activity for cause-specific mortality (CVD and cancer) and overcomes important limitations of these previous 2 studies by assessing longer-term LTPA trajectories from adolescence through adulthood in a larger sample of US adults, with 22 219 CVD and 16 388 cancer deaths.

### Limitations

The present study is not without limitations. First, information about participation in LTPA was collected at the time of study enrollment using historical questions about LTPA. Historical items about lifestyle behaviors may be susceptible to systematic (eg, social desirability) and random reporting errors. Those sources of error in prospective studies are generally expected to result in attenuation of the strength of associations (ie, bias to the null).^30^ We examined the random error associated with our measure of LTPA in a parallel study that included approximately 900 adult AARP members and found adequate reliability (intraclass correlations ranged from 0.5 to 0.6 when assessed 6 months apart). The reliability of our historical LTPA measures was consistent with previous reliability studies of historical items for physical activity, with correlations ranging from 0.4 to 0.8.^31,32,33,34,35^ The validity of historical items to assess life course participation in LTPA has been less studied given the challenges in assembling a study of this nature. However, a study by Besson et al^36^ also examined the validity of historical reports of physical activity for the previous 7 to 13 years and found these to be correlated by 0.4 against concurrent estimates obtained from device-based measures. These studies and our own examinations of reliability suggest that our measures of LTPA may be acceptable for our purposes. A second limitation was that our estimates of LTPA duration (hours per week) should be interpreted as approximations rather than as specific duration values. For our analysis, we converted a categorical indicator of LTPA duration into continuous values in the process of modeling trajectories across the 4 time periods. For example, participants’ responses indicating that they spent 4 to 7 hours or spent more than 7 hours per week in LTPA were recoded as 5.5 and 9 hours per week, respectively. Thus, the absolute duration of LTPA associated with each trajectory should be interpreted with this in mind. Another limitation is that it is not clear whether our results can be generalized to adults who are outside the primary demographic and health characteristics of the NIH-AARP cohort. Our analytical sample included approximately 300 000 participants from the 539 213 participants who were free of colorectal cancer, breast cancer, prostate cancer, or renal disease at the time of enrollment and hence eligible for the study. However, our additional examinations showed that the distributions of key demographic and lifestyle characteristics were similar between the full NIH-AARP sample and our analytical sample, minimizing possible bias due to nonresponse. The NIH-AARP study is observational, and while its prospective design establishes temporal sequence between LTPA and mortality, we cannot rule out unmeasured confounding and reverse causality. Health status at baseline could have influenced our trajectories and associations with mortality; however, our results remained consistent after we adjusted our models for many risk factors. Our examinations in sensitivity analyses also did not reveal evidence of reverse causality, as illustrated by similar HRs after adjusting or excluding participants with health conditions at baseline, or effect modification associated with BMI, or LTPA trajectory misclassification. Ideally, a randomized controlled trial could overcome these limitations; but, such a study would be infeasible. Future studies are needed to examine whether our findings will hold in a more ethnically and economically diverse sample. Long-term participation in LTPA can also produce health benefits that go beyond mortality; hence, additional studies are also needed to explore how the health pathways through each LTPA trajectory might act to lower the risk for mortality.

## Conclusions

Increasing LTPA later in adulthood was associated with mortality benefits that were similar to those associated with maintaining higher levels of LTPA across the adult life course. Our findings suggest that it is not too late for adults to become active. These findings are particularly informative for health care professionals advising individuals who have been physically inactive throughout much of their adulthood that substantial health benefits can still be gained by improving their physical activity habits.

## Notes

Supplement. **eTable 1.** Complete Distributions of Categorical Covariates Amongst Participants Belonging to the ‘Maintainers’ Trajectories (of Leisure-Time Physical Activity) **eTable 2.** Complete Distributions of Categorical Covariates Amongst Participants Belonging to the ‘Increasers’ or ‘Decreasers’ Trajectories (of Leisure-Time Physical Activity) **eTable 3.** Hazard Ratios (95% Confidence Intervals) for All-Cause, Cardiovascular Disease (CVD), and Cancer Mortality for Leisure-Time Physical Activity Maintainers **eTable 4.** Hazard Ratios (95% Confidence Intervals) for All-Cause, Cardiovascular Disease (CVD), and Cancer Mortality for Leisure-Time Physical Activity Trajectory Groups ‘Increasers’ and ‘Decreasers’ **eTable 5.** Hazard Ratios (95% Confidence Intervals) Among the Trajectory Groups ‘Maintainers’ After Excluding Early Deaths (First Two Years) **eTable 6.** Hazard Ratios (95% Confidence Intervals) Among the Trajectory Groups ‘Increasers’ and ‘Decreasers’ After Excluding Early Deaths (First Two Years) **eTable 7.** Hazard Ratios (95% Confidence Intervals) Among Trajectory Groups ‘Maintainers’ After Excluding Individuals With Trajectory Assignment Probabilities Lower Than 80% **eTable 8.** Hazard Ratios (95% Confidence Intervals) Among Increasers and Decreasers After Excluding Individuals With Trajectory Assignment Probabilities Lower Than 80% **eFigure 1.** Item Included in the AARP Risk Factor Questionnaire to Assess Participation in Leisure-Time Physical Activity at Ages 15-18 Years, 19-29 Years, 35-39 Years, and in the Previous 10 Years (Assumed as 40-61 Years) **eFigure 2.** Leisure-Time Physical Activity (LTPA) Trajectories and Respective Hazard Ratios (95% Confidence Intervals) for All-Cause Mortality Among Maintainers (A), Increasers (B), and Decreasers (C) **eFigure 3.** Leisure-Time Physical Activity (LTPA) Trajectories and Respective Hazard Ratios (95% Confidence Intervals) for Men (n = 183,453) and Women (n = 131,606) **eFigure 4.** Leisure-Time Physical Activity (LTPA) Trajectories and Respective Hazard Ratios (95% Confidence Intervals) for All-Cause Mortality Among Maintainers (A), Increasers (B), and Decreasers (C)
