# Supplementary material for: Association of Leisure-Time Physical Activity Across the Adult Life Course With All-Cause and Cause-Specific Mortality
Source: JAMA Netw Open. 2019 Mar 8;2(3):e190355. doi: 10.1001/jamanetworkopen.2019.0355 (PMC6484624; doi:10.1001/jamanetworkopen.2019.0355)
Supplement: Supplement. — eTable 1. Complete Distributions of Categorical Covariates Amongst Participants Belonging to the ‘Maintainers’ Trajectories (of Leisure-Time Physical Activity) eTable 2. Complete Distributions of Categorical Covariates Amongst Participants Belonging to the ‘Increasers’ or ‘Decreasers’ Trajectories (of Leisure-Time Physical Activity) eTable 3. Hazard Ratios (95% Confidence Intervals) for All-Cause, Cardiovascular Disease (CVD), and Cancer Mortality for Leisure-Time Physical Activity Maintainers eTable 4. Hazard Ratios (95% Confidence Intervals) for All-Cause, Cardiovascular Disease (CVD), and Cancer Mortality for Leisure-Time Physical Activity Trajectory Groups ‘Increasers’ and ‘Decreasers’ eTable 5. Hazard Ratios (95% Confidence Intervals) Among the Trajectory Groups ‘Maintainers’ After Excluding Early Deaths (First Two Years) eTable 6. Hazard Ratios (95% Confidence Intervals) Among the Trajectory Groups ‘Increasers’ and ‘Decreasers’ After Excluding Early Deaths (First Two Years) eTable 7. Hazard Ratios (95% Confidence Intervals) Among Trajectory Groups ‘Maintainers’ After Excluding Individuals With Trajectory Assignment Probabilities Lower Than 80% eTable 8. Hazard Ratios (95% Confidence Intervals) Among Increasers and Decreasers After Excluding Individuals With Trajectory Assignment Probabilities Lower Than 80% eFigure 1. Item Included in the AARP Risk Factor Questionnaire to Assess Participation in Leisure-Time Physical Activity at Ages 15-18 Years, 19-29 Years, 35-39 Years, and in the Previous 10 Years (Assumed as 40-61 Years) eFigure 2. Leisure-Time Physical Activity (LTPA) Trajectories and Respective Hazard Ratios (95% Confidence Intervals) for All-Cause Mortality Among Maintainers (A), Increasers (B), and Decreasers (C) eFigure 3. Leisure-Time Physical Activity (LTPA) Trajectories and Respective Hazard Ratios (95% Confidence Intervals) for Men (n = 183,453) and Women (n = 131,606) eFigure 4. Leisure-Time Physical Activity (LTPA) Trajectories and Respective Ha [file jamanetwopen-2-e190355-s001.pdf]

Supplementary Online Content

Saint-Maurice PF, Coughlan D, Kelly SP, et al. Association of leisure-time physical activity across the adult life course with all-cause and cause-specific mortality. *JAMA Netw Open*. 2019;2(3):e190355. doi:10.1001/jamanetworkopen.2019.0355

eTable 1. Complete Distributions of Categorical Covariates Amongst Participants Belonging to the 'Maintainers' Trajectories (of Leisure-Time Physical Activity)

eTable 2. Complete Distributions of Categorical Covariates Amongst Participants Belonging to the 'Increasers' or 'Decreasers' Trajectories (of Leisure-Time Physical Activity)

eTable 3. Hazard Ratios (95% Confidence Intervals) for All-Cause, Cardiovascular Disease (CVD), and Cancer Mortality for Leisure-Time Physical Activity Maintainers

eTable 4. Hazard Ratios (95% Confidence Intervals) for All-Cause, Cardiovascular Disease (CVD), and Cancer Mortality for Leisure-Time Physical Activity Trajectory Groups 'Increasers' and 'Decreasers'

eTable 5. Hazard Ratios (95% Confidence Intervals) Among the Trajectory Groups 'Maintainers' After Excluding Early Deaths (First Two Years)

eTable 6. Hazard Ratios (95% Confidence Intervals) Among the Trajectory Groups 'Increasers' and 'Decreasers' After Excluding Early Deaths (First Two Years)

eTable 7. Hazard Ratios (95% Confidence Intervals) Among Trajectory Groups 'Maintainers' After Excluding Individuals With Trajectory Assignment Probabilities Lower Than 80%

eTable 8. Hazard Ratios (95% Confidence Intervals) Among Increasers and Decreasers After Excluding Individuals With Trajectory Assignment Probabilities Lower Than 80%

eFigure 1. Item Included in the AARP Risk Factor Questionnaire to Assess Participation in Leisure-Time Physical Activity at Ages 15-18 Years, 19-29 Years, 35-39 Years, and in the Previous 10 Years (Assumed as 40-61 Years)

eFigure 2. Leisure-Time Physical Activity (LTPA) Trajectories and Respective Hazard Ratios (95% Confidence Intervals) for All-Cause Mortality Among Maintainers (A), Increasers (B), and Decreasers (C)

© 2019 Saint-Maurice PF et al. *JAMA Network Open*.

eFigure 3. Leisure-Time Physical Activity (LTPA) Trajectories and Respective Hazard Ratios (95% Confidence Intervals) for Men (n=183,453) and Women (n=131,606)

eFigure 4. Leisure-Time Physical Activity (LTPA) Trajectories and Respective Hazard Ratios (95% Confidence Intervals) for All-Cause Mortality Among Maintainers (A), Increasers (B), and Decreasers (C)

This supplementary material has been provided by the authors to give readers additional information about their work.

© 2019 Saint-Maurice PF et al. *JAMA Network Open*.

eTable 1. Complete Distributions of Categorical Covariates Amongst Participants Belonging to the 'Maintainers' Trajectories (of Leisure-Time Physical Activity)

Trajectory	1 (REF)	5	7	8	10
N	13,170	48,195	72,783	32,608	9,898
	**N (%)**	**N (%)**	**N (%)**	**N (%)**	**N (%)**
Males	6,588, (50.0)	24,963 (51.8)	42,868 (58.9)	21,875 (67.1)	7,347 (74.2)
Females	6,582 (50.0)	23,232 (48.2)	29,915 (41.1)	10,733 (32.9)	2,551 (25.8)
BMI - at 18 years					
BMI < 25.0 kg/m^2	10,049 (76.3)	38,878 (80.7)	60,506 (83.1)	27,479 (84.3)	8,391 (84.8)
25.0 ≤ BMI < 30.0 kg/m^2	1,203 (9.1)	4,039 (8.4)	6,094 (8.4)	2,868 (8.8)	928 (9.4)
BMI ≥ 30.0 kg/m^2	357 (2.7)	942 (2.0)	780 (1.1)	358 (1.1)	102 (1.0)
Unknown	1,561 (11.9)	4,336 (9.0)	5,403 (7.4)	1,903 (5.8)	477 (4.8)
BMI - current					
BMI < 25.0 kg/m^2	4,257 (32.3)	17,037 (35.4)	28,030 (38.5)	13,233 (40.6)	4,279 (43.2)
25.0 ≤ BMI < 30.0 kg/m^2	4,925 (37.4)	19,036 (39.5)	31,122 (42.8)	14,212 (43.6)	4,181 (42.2)
BMI ≥ 30.0 kg/m^2	3,611 (27.4)	11,052 (22.9)	12,252 (16.8)	4,627 (14.2)	1,285 (13.0)
Unknown	377 (2.9)	1,070 (2.2)	1,379 (1.9)	536 (1.6)	153 (1.6)
Race					
Non-Hispanic White	11,840 (89.9)	44,164 (91.6)	68,154 (93.6)	30,742 (94.3)	9,342 (94.4)
Non-Hispanic Black	618 (4.7)	1,922 (4.0)	1,840 (2.5)	723 (2.2)	219 (2.2)
Hispanic	272 (2.1)	828 (1.7)	1,068 (1.5)	448 (1.4)	133 (1.3)
Other	292 (2.2)	760 (1.6)	895 (1.2)	390 (1.2)	126 (1.3)
Unknown	148 (1.1)	521 (1.1)	826 (1.1)	305 (0.9)	78 (0.8)
Education					
<8 years	230 (1.8)	249 (0.5)	301 (0.4)	76 (0.2)	16 (0.2)
8-11 years	1,105 (8.4)	2,422 (5.0)	3,159 (4.3)	876 (2.7)	208 (2.1)

© 2019 Saint-Maurice PF et al. *JAMA Network Open*.

High-school	3,484 (26.5)	10,239 (21.2)	13,666 (18.8)	4,660 (14.3)	1,227 (12.4)
Post high-school	1,254 (9.5)	4,735 (9.8)	7,715 (10.6)	2,727 (8.4)	813 (8.2)
Some college	2,614 (19.9)	10,629 (22.1)	17,724 (24.4)	7,295 (22.4)	2,045 (20.7)
College graduate	1,952 (14.8)	8,773 (18.2)	14,131 (19.4)	7,478 (22.9)	2,470 (25.0)
Post-graduate	2,170 (16.5)	9,946 (20.6)	14,129 (19.4)	8,812 (27.0)	2,929 (29.6)
Unknown	361 (2.7)	1,202 (2.5)	1,958 (2.7)	684 (2.1)	190 (1.9)
Smoking status/dose					
Never smoked	5,175 (39.3)	18,259 (37.9)	26,216 (36.0)	11,682 (35.8)	3,137 (31.7)
Quit: ≤20 cigs/day	3,090 (23.5)	13,141 (27.3)	20,892 (28.7)	9,865 (30.3)	3,067 (31.0)
Quit: >20 cigs/day	2,684 (20.4)	9,754 (20.2)	14,924 (20.5)	7,379 (22.6)	2,807 (28.4)
Current smoker: ≤20 cigs/day	1,041 (7.9)	3,552 (7.4)	5,573 (7.7)	1,771 (5.4)	408 (4.1)
Current smoker: >20 cigs/day	645 (4.9)	1,889 (3.9)	2,815 (3.9)	887 (2.7)	165 (1.7)
Unknown	535 (4.1)	1600 (3.3)	2,363 (3.3)	1,024 (3.1)	314 (3.2)
Vitamin/mineral suppl.					
No	4,404 (33.4)	13,783 (28.6)	19,781 (27.2)	8,448 (25.9)	2,465 (24.9)
Yes, < once/month	743 (5.6)	2,566 (5.3)	3,387 (4.7)	1,583 (4.9)	424 (4.3)
Yes, once/month or more	7,454 (56.6)	30,115 (62.5)	46,902 (64.4)	21,528 (66.0)	6,719 (67.9)
Unknown	569 (4.3)	1,731 (3.59)	2,713 (3.7)	1,050 (3.2)	290 (2.9)

Abbreviations: REF: referent group; BMI: body mass index; cigs: cigarettes; suppl: supplementation

© 2019 Saint-Maurice PF et al. *JAMA Network Open*.

eTable 2. Complete Distributions of Categorical Covariates Amongst Participants Belonging to the 'Increasers' or 'Decreasers' Trajectories (of Leisure-Time Physical Activity)

	Increasers		Decreasers		
Trajectory	2	3	4	6	9
N	25,094	16,099	16,611	35,846	44,755
	**N (%)**	**N (%)**	**N (%)**	**N (%)**	**N (%)**
Males	13,675 (54.5)	5,677 (35.3)	7,155 (43.1)	22,492 (62.8)	30,811 (68.8)
Females	11,419 (45.5)	10,422 (67.7)	9,456 (56.9)	13,352 (37.3)	13,944 (31.2)
BMI - at 18 years					
BMI < 25.0 kg/m^2	20,575 (82.0)	13,173 (81.8)	13,724 (82.6)	29,468 (82.2)	37,051 (82.8)
25.0 ≤ BMI < 30.0 kg/m^2	2,101 (8.4)	1,076 (6.7)	1,270 (7.7)	3,508 (9.8)	4,334 (9.7)
BMI ≥ 30.0 kg/m^2	464 (1.9)	247 (1.5)	275 (1.7)	552 (1.5)	736 (1.6)
Unknown	1,954 (7.8)	1,603 (10.0)	1,342 (8.1)	2,318 (6.5)	2,634 (5.9)
BMI - current					
BMI < 25.0 kg/m^2	11,997 (47.8)	7,542 (46.9)	5,587 (33.6)	9,441 (26.3)	13,047 (29.2)
25.0 ≤ BMI < 30.0 kg/m^2	9,615 (38.3)	5,799 (36.0)	6,582 (39.6)	14,986 (41.8)	19.173 (42.8)
BMI ≥ 30.0 kg/m^2	3,015 (12.0)	2,406 (15.0)	4,124 (24.8)	10,762 (30.0)	11,783 (26.3)
Unknown	467 (1.9)	352 (2.2)	318 (1.9)	657 (1.8)	752 (1.7)
Race					
Non-Hispanic White	23,390 (93.2)	15,045 (93.5)	15,499 (93.3)	33,161 (92.5)	41,312 (92.3)
Non-Hispanic Black	597 (2.4)	447 (2.8)	514 (3.1)	1,353 (3.8)	1,554 (3.5)
Hispanic	423 (1.7)	250 (1.6)	243 (1.5)	500 (1.4)	760 (1.7)
Other	414 (1.7)	183 (1.1)	186 (1.1)	406 (1.1)	681 (1.5)
Unknown	270 (1.1)	174 (1.1)	169 (1.0	426 (1.2)	448 (1.0)
Education					
<8 years	104 (0.4)	80 (0.5)	58 (0.4)	162 (0.5)	118 (0.3)

© 2019 Saint-Maurice PF et al. *JAMA Network Open*.

8-11 years	1,032 (4.1)	822 (5.1)	737 (4.4)	1,355 (3.8)	1,420 (3.2)
High-school	4,486 (17.9)	4,026 (25.0)	3,275 (19.7)	5,738 (16.0)	6,875 (15.4)
Post high-school	2,211 (8.8)	1,750 (10.9)	1,929 (11.6)	3,792 (10.6)	4,261 (9.5)
Some college	5,447 (21.7)	3,693 (22.9)	4,227 (25.5)	9,591 (26.8)	10,600 (23.7)
College graduate	5,108 (20.4)	2,702 (16.8)	2,961 (17.8)	7,116 (19.9)	9,830 (22.0)
Post-graduate	6,137 (24.5)	2,627 (16.3)	2,987 (18.0)	7,196 (20.1)	10,708 (23.9)
Unknown	569 (2.3)	399 (2.5)	437 (2.6)	896 (2.5)	943 (2.1)
Smoking status/dose					
Never smoked	9,129 (36.4)	6,653 (41.3)	5,979 (36.0)	10,966 (30.6)	14,861 (33.2)
Quit: ≤20 cigs/day	7,768 (31.0)	4,661 (29.0)	4,536 (27.3)	9,317 (26.0)	12,141 (27.1)
Quit: >20 cigs/day	5,741 (22.9)	2,663 (16.5)	3,359 (20.2)	9,223 (25.7)	10,897 (24.4)
Current smoker: ≤20 cigs/day	1,155 (4.6)	1,162 (7.2)	1,445 (8.7)	3.090 (8.6)	3,283 (7.3)
Current smoker: >20 cigs/day	453 (1,8)	455 (2.8)	786 (4.7)	2,137 (6.0)	2,207 (4.9)
Unknown	848 (3.4)	505 (3.1)	506 (3.1)	1,113 (3.1)	1,366 (3.1)
Vitamin/mineral supp.					
No	5,785 (23.1)	3,847 (23.9)	4,416 (26.6)	10,538 (29.4)	13,366 (29.9)
Yes, < once/month	1,120 (4.5)	752 (4.7)	878 (5.3)	1,898 (5.3)	2,391 (5.3)
Yes, once/month or more	17,291 (68.9)	10,893 (67.7)	10,745 (64.7)	22,220 (62.0)	27,547 (61.6)
Unknown	898 (3.6)	607 (3.77)	572 (3.44)	1,190 (3.32)	1,451 (3.3)

Abbreviations: REF: referent group; BMI: body mass index; cigs: cigarettes; suppl: supplementation

© 2019 Saint-Maurice PF et al. *JAMA Network Open*.

eTable 3. Hazard Ratios (95% Confidence Intervals) for All-Cause, Cardiovascular Disease (CVD), and Cancer Mortality for Leisure-Time Physical Activity Maintainers

	**Maintainers**				
Trajectory	1 (REF)	5	7	8	10
N	13,170	48,195	72,783	32,608	9,898
All-cause Deaths	3,789	11,214	15,494	6,241	1,874
Model 1	1.00	**0.79 (0.77, 0.82)**	**0.66 (0.64, 0.69)**	**0.58 (0.66, 0.60)**	**0.55 (0.52, 0.58)**
Model 2	1.00	**0.84 (0.81, 0.87)**	**0.70 (0.67, 0.72)**	**0.65 (0.62, 0.68)**	**0.63 (0.60, 0.67)**
Model 3	1.00	**0.84 (0.81, 0.87)**	**0.71 (0.68, 0.73)**	**0.66 (0.63, 0.68)**	**0.64 (0.60, 0.68)**
CVD Deaths	1,237	3,545	4,755	1,850	579
Model 1	1.00	**0.77 (0.72, 0.82)**	**0.61 (0.58, 0.66)**	**0.51 (0.48, 0.55)**	**0.50 (0.46, 0.55)**
Model 2	1.00	**0.81 (0.76, 0.87)**	**0.65 (0.61, 0.69)**	**0.57 (0.53, 0.62)**	**0.57 (0.52, 0.63)**
Model 3	1.00	**0.82 (0.77, 0.88)**	**0.66 (0.62, 0.70)**	**0.58 (0.54, 0.63)**	**0.58 (0.53, 0.64)**
Cancer Deaths	744	2,519	3,790	1,540	477
Model 1	1.00	**0.91 (0.84, 0.99)**	**0.84 (0.78, 0.91)**	**0.75 (0.69, 0.82)**	**0.75 (0.67, 0.84)**
Model 2	1.00	0.99 (0.88, 1.04)	**0.89 (0.82, 0.97)**	**0.85 (0.77, 0.92)**	**0.86 (0.76, 0.96)**
Model 3	1.00	0.96 (0.88, 1.04)	**0.90 (0.83, 0.97)**	**0.85 (0.78, 0.93)**	**0.86 (0.77, 0.97)**

Model 1: Age + sex
Model 2: Model 1 + education + race + smoking status/dose + total energy intake + diet percent fat + alcohol consumption + red meat consumption + consumption of fruits + consumption of vegetables + vitamin/mineral supplementation
Model 3: Model 2 + body mass index at age 18 years
Abbreviations: REF: Referent group

© 2019 Saint-Maurice PF et al. *JAMA Network Open*.

eTable 4. Hazard Ratios (95% Confidence Intervals) for All-Cause, Cardiovascular Disease (CVD), and Cancer Mortality for Leisure-Time Physical Activity Trajectory Groups 'Increasers' and 'Decreasers'

	**Maintainers**	**Increasers**		**Decreasers**		
Trajectory	1 (REF)	2	3	4	6	9
N	13,170	25,094	16,099	16,611	35,846	44,755
All-cause Deaths	3,789	4,592	2,969	4,319	10,138	10,747
Model 1	1.00	**0.57 (0.55, 0.59)**	**0.62 (0.59, 0.65)**	**0.89 (0.85, 0.93)**	0.96 (0.93, 1.00)	**0.82 (0.79, 0.85)**
Model 2	1.00	**0.64 (0.62, 0.67)**	**0.68 (0.64, 0.71)**	**0.91 (0.87, 0.95)**	**0.95 (0.92, 0.99)**	**0.86 (0.83, 0.89)**
Model 3	1.00	**0.65 (0.62, 0.68)**	**0.68 (0.65, 0.72)**	**0.92 (0.88, 0.96)**	0.96 (0.92, 1.00)	**0.86 (0.83, 0.90)**
CVD Deaths	1,237	1,345	810	1,321	3,293	3,484
Model 1	1.00	**0.50 (0.47, 0.55)**	**0.53 (0.49, 0.58)**	**0.84 (0.78, 0.91)**	0.94 (0.88, 1.01)	**0.81 (0.75, 0.86)**
Model 2	1.00	**0.56 (0.52, 0.61)**	**0.57 (0.53, 0.63)**	**0.87 (0.80, 0.94)**	0.95 (0.89, 1.01)	**0.85 (0.80, 0.91)**
Model 3	1.00	**0.57 (0.53, 0.61)**	**0.58 (0.53, 0.64)**	**0.88 (0.81, 0.95)**	0.96 (0.90, 1.03)	**0.86 (0.81, 0.92)**
Cancer Deaths	744	1,134	716	906	2,098	2,464
Model 1	1.00	**0.73 (0.67, 0.80)**	**0.76 (0.68, 0.84)**	0.95 (0.86, 1.04)	1.02 (0.94, 1.11)	0.97 (0.89, 1.05)
Model 2	1.00	**0.84 (0.77, 0.92)**	**0.83 (0.75, 0.92)**	0.96 (0.87, 1.05)	0.99 (0.91, 1.08)	0.99 (0.92, 1.08)
Model 3	1.00	**0.84 (0.77, 0.92)**	**0.83 (0.75, 0.93)**	0.96 (0.87, 1.06)	0.99 (0.91, 1.08)	0.99 (0.92, 1.08)

Model 1: Age + sex

Model 2: Model 1 + education + race + smoking status/dose + total energy intake + diet percent fat + alcohol consumption + red meat consumption + consumption of fruits + consumption of vegetables + vitamin/mineral supplementation

Model 3: Model 2 + body mass index at age 18 years

Abbreviations: REF: Referent group

© 2019 Saint-Maurice PF et al. *JAMA Network Open*.

eTable 5. Hazard Ratios (95% Confidence Intervals) Among the Trajectory Groups 'Maintainers' After Excluding Early Deaths (First Two Years)

	Maintainers				
Trajectory	1 (REF)	5	7	8	10
N	12,861	47,350	71,844	32,329	9,783
% of initial sample[1]	97.7	98.2	98.7	99.1	98.8
All-cause Deaths	3,488	10,393	14,598	5,885	1,763
Model 3	1.00	**0.85 (0.81, 0.88)**	**0.72 (0.69, 0.75)**	**0.67 (0.64, 0.70)**	**0.65 (0.61, 0.69)**
CVD Deaths	1,136	3,246	4,414	1,714	545
Model 3	1.00	**0.81 (0.76, 0.87)**	**0.66 (0.62, 0.70)**	**0.58 (0.54, 0.63)**	**0.59 (0.53, 0.66)**
Cancer Deaths	662	2,314	3,542	1,430	437
Model 3	1.00	0.99 (0.91, 1.08)	0.94 (0.86, 1.02)	**0.88 (0.80, 0.97)**	0.88 (0.78, 1.00)

[1](Number of participants after exclusions/Number of participants on original sample) X 100

Model 3: Age + Sex + + education + race + smoking status/dose + total energy intake + diet percent fat + alcohol consumption + red meat consumption + consumption of fruits + consumption of vegetables + vitamin/mineral supplementation + body mass index at age 18 years

Abbreviations: REF: Referent group

© 2019 Saint-Maurice PF et al. *JAMA Network Open*.

eTable 6. Hazard Ratios (95% Confidence Intervals) Among the Trajectory Groups 'Increasers' and 'Decreasers' After Excluding Early Deaths (First Two Years)

	**Maintainers**	**Increasers**		**Decreasers**		
Trajectory	1 (REF)	2	3	4	6	9
N	12,861	24,828	15,919	16,271	35,048	43,914
% of initial sample1	97.7	98.9	98.9	98.0	97.8	98.1
All-cause Deaths	1,784	4,343	2,797	3,991	9,368	9,932
Model 3	1.00	**0.66 (0.63, 0.69)**	**0.69 (0.66, 0.73)**	**0.92 (0.88, 0.96)**	0.96 (0.93, 1.00)	**0.87 (0.83, 0.90)**
CVD Deaths	1,136	1,261	751	1,202	3,014	3,156
Model 3	1.00	**0.57 (0.53, 0.62)**	**0.58 (0.53, 0.63)**	**0.86 (0.80, 0.94)**	0.96 (0.89, 1.03)	**0.85 (0.79, 0.91)**
Cancer Deaths	424	1,049	666	828	1,928	2,269
Model 3	1.00	**0.87 (0.79, 0.96)**	**0.86 (0.78, 0.96)**	0.98 (0.89, 1.09)	1.03 (0.94, 1.12)	1.03 (0.94, 1.12)

[1](Number of participants after exclusions/Number of participants on original sample) X 100

Model 3: Age + Sex + + education + race + smoking status/dose + total energy intake + diet percent fat + alcohol consumption + red meat consumption + consumption of fruits + consumption of vegetables + vitamin/mineral supplementation + body mass index at age 18 years

Abbreviations: REF: Referent group

© 2019 Saint-Maurice PF et al. *JAMA Network Open*.

eTable 7. Hazard Ratios (95% Confidence Intervals) Among Trajectory Groups 'Maintainers' After Excluding Individuals With Trajectory Assignment Probabilities Lower Than 80%

	**Maintainers**				
Trajectory	1 (REF)	5	7	8	10
N	11,738	45,288	60,205	25,177	8,835
% of initial sample[1]	89.1	94.0	82.7	77.2	89.3
All-cause Deaths	3393	10,466	12,948	4,819	1,701
Model 3	1.00	**0.83 (0.80, 0.86)**	**0.70 (0.68, 0.73)**	**0.65 (0.62, 0.68)**	**0.64 (0.61, 0.68)**
CVD Deaths	1104	3,302	3,950	1,456	526
Model 3	1.00	**0.81 (0.76, 0.87)**	**0.65 (0.61, 0.70)**	**0.59 (0.54, 0.64)**	**0.59 (0.53, 0.65)**
Cancer Deaths	671	2,357	3,157	1,193	431
Model 3	1.00	0.94 (0.86, 1.03)	**0.88 (0.81, 0.96)**	**0.83 (0.76, 0.92)**	**0.85 (0.76, 0.97)**

[1](Number of participants after exclusions/Number of participants on original sample) X 100

Model 3: Age + Sex + + education + race + smoking status/dose + total energy intake + diet percent fat + alcohol consumption + red meat consumption + consumption of fruits + consumption of vegetables + vitamin/mineral supplementation + body mass index at age 18 years

Abbreviations: REF: Referent group

© 2019 Saint-Maurice PF et al. *JAMA Network Open*.

eTable 8. Hazard Ratios (95% Confidence Intervals) Among Increasers and Decreasers After Excluding Individuals With Trajectory Assignment Probabilities Lower Than 80%

		**Increasers**		**Decreasers**		
Trajectory	1 (REF)	2	3	4	6	9
N	11,738	23,760	11,859	6,979	35,472	41,124
% of initial sample[1]	89.1	94.7	73.7	42.0	99.0	91.9
All-cause Deaths	3,393	4,307	2,198	1,897	10,056	9,891
Model 3	1.00	**0.64 (0.61, 0.67)**	**0.67 (0.64, 0.71)**	**0.96 (0.91, 1.02)**	**0.96 (0.92, 0.99)**	**0.86 (0.83, 0.89)**
CVD Deaths	1,104	1,248	589	551	3,268	3,196
Model 3	1.00	**0.56 (0.51, 0.60)**	**0.56 (0.51, 0.62)**	**0.89 (0.80, 0.99)**	0.96 (0.90, 1.03)	**0.86 (0.80, 0.92)**
Cancer Deaths	671	1,070	532	399	2,078	2,274
Model 3	1.00	**0.83 (0.75, 0.91)**	**0.82 (0.73, 0.92)**	1.00 (0.88, 1.13)	0.98 (0.90, 1.07)	0.98 (0.89, 1.07)

[1](Number of participants after exclusions/Number of participants on original sample) X 100

Model 3: Age + Sex + education + race + smoking status/dose + total energy intake + diet percent fat + alcohol consumption + red meat consumption + consumption of fruits + consumption of vegetables + vitamin/mineral supplementation + body mass index at age 18 years

Abbreviations: REF: Referent group

© 2019 Saint-Maurice PF et al. *JAMA Network Open*.

55. **Read the list of examples of moderate and vigorous activities in the box below.**

EXAMPLES OF MODERATE AND VIGOROUS ACTIVITIES:

Tennis	Heavy gardening	Cheerleading/drill team	Rowing
Golf (walking)	Weight lifting	Handball/racquetball	Aerobics
Biking	Basketball/baseball	Hiking/climbing mountains	Jogging/running
Swimming	Football/soccer	Fast walking/fast dancing	Heavy housework

Think back to the ages and times listed in the table below. Mark the circles that best describe how often you participated in moderate and vigorous activities at the ages and time listed. DO NOT INCLUDE ACTIVITIES THAT YOU REPORTED IN QUESTIONS 48 - 51 ON PAGE 13.

How often did you participate in moderate and vigorous activities at the following ages and time?	HOW OFTEN (MARK ONLY ONE RESPONSE)					
	Never	Rarely	Weekly, but less than 1 hour per week	1-3 hours per week	4-7 hours per week	More than 7 hours per week
15-18 years old	○	○	○	○	○	○
19-29 years old	○	○	○	○	○	○
35-39 years old	○	○	○	○	○	○
in the past 10 years	○	○	○	○	○	○

eFigure 1. Item Included in the AARP Risk Factor Questionnaire to Assess Participation in Leisure-Time Physical Activity at Ages 15-18 Years, 19-29 Years, 35-39 Years, and in the Previous 10 Years (Assumed as 40-61 Years).

© 2019 Saint-Maurice PF et al. *JAMA Network Open*.

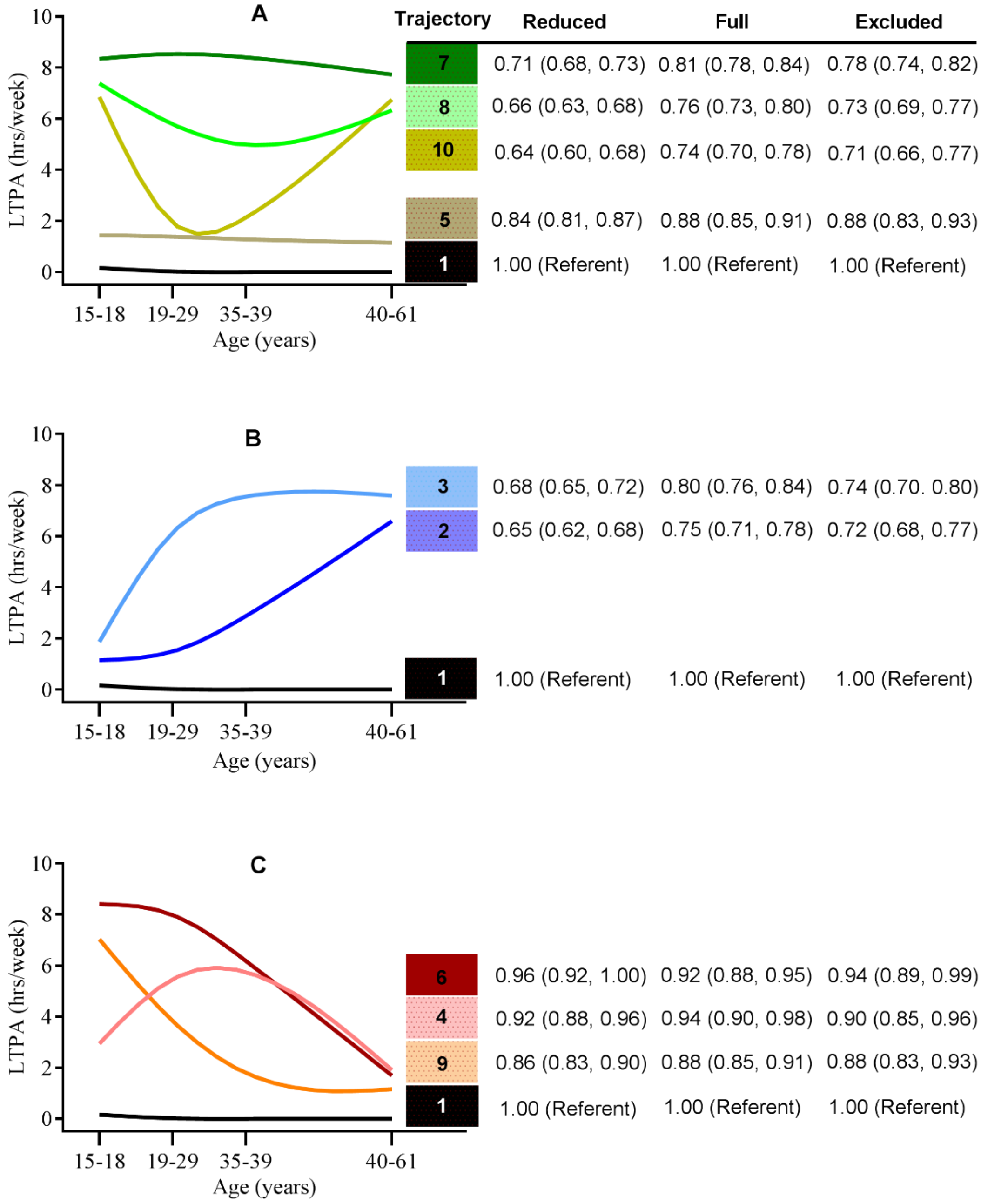

	Hazard ratios (95% CI)		
Trajectory	Reduced	Full	Excluded
7	0.71 (0.68, 0.73)	0.81 (0.78, 0.84)	0.78 (0.74, 0.82)
8	0.66 (0.63, 0.68)	0.76 (0.73, 0.80)	0.73 (0.69, 0.77)
10	0.64 (0.60, 0.68)	0.74 (0.70, 0.78)	0.71 (0.66, 0.77)
5	0.84 (0.81, 0.87)	0.88 (0.85, 0.91)	0.88 (0.83, 0.93)
1	1.00 (Referent)	1.00 (Referent)	1.00 (Referent)
3	0.68 (0.65, 0.72)	0.80 (0.76, 0.84)	0.74 (0.70. 0.80)
2	0.65 (0.62, 0.68)	0.75 (0.71, 0.78)	0.72 (0.68, 0.77)
1	1.00 (Referent)	1.00 (Referent)	1.00 (Referent)
6	0.96 (0.92, 1.00)	0.92 (0.88, 0.95)	0.94 (0.89, 0.99)
4	0.92 (0.88, 0.96)	0.94 (0.90, 0.98)	0.90 (0.85, 0.96)
9	0.86 (0.83, 0.90)	0.88 (0.85, 0.91)	0.88 (0.83, 0.93)
1	1.00 (Referent)	1.00 (Referent)	1.00 (Referent)

eFigure 2. Leisure-Time Physical Activity (LTPA) Trajectories and Respective Hazard Ratios (95% Confidence Intervals) for All-Cause Mortality Among Maintainers (A), Increasers (B), and Decreasers (C). "Reduced" model hazard ratios were computed using model 3 and are not adjusted for disease conditions. Covariates include: age, sex, education, race, smoking status/dose, total energy intake, diet percent fat, alcohol consumption, red meat consumption, consumption of fruits, consumption of vegetables, vitamin/mineral supplementation, body mass

© 2019 Saint-Maurice PF et al. *JAMA Network Open*.

index at age 18 years. “Full” model hazard ratios include all covariates listed above and are further adjusted for prevalent conditions reported at age 50-71 years (i.e., baseline), including: heart disease, cancer, diabetes, stroke, emphysema, and overall health status. “Excluded” hazard ratios does not include participants with reported health conditions at age 50-71 years (heart disease, cancer, diabetes, stroke, emphysema, and overall health status) and covariates include: age, sex, education, race, smoking status/dose, total energy intake, diet percent fat, alcohol consumption, red meat consumption, consumption of fruits, consumption of vegetables, vitamin/mineral supplementation and, body mass index at age 18 years.

© 2019 Saint-Maurice PF et al. *JAMA Network Open*.

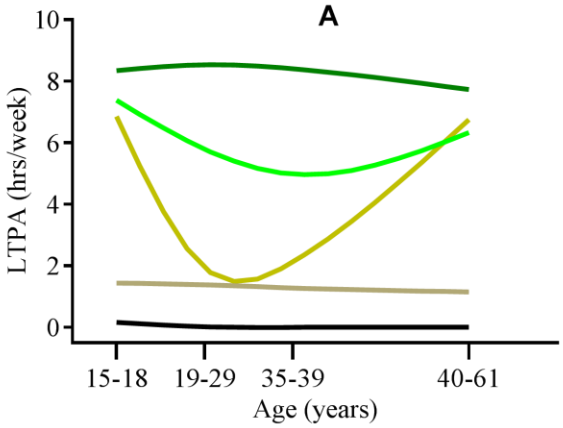

Trajectory	Hazard ratios (95% CI) Men	Women
7	0.70 (0.67, 0.73)	0.73 (0.69, 0.78)
8	0.65 (0.61, 0.68)	0.69 (0.65, 0.74)
10	0.64 (0.60, 0.68)	0.64 (0.57, 0.72)
5	0.85 (0.81, 0.90)	0.83 (0.78, 0.88)
1	1.00 (Referent)	1.00 (Referent)

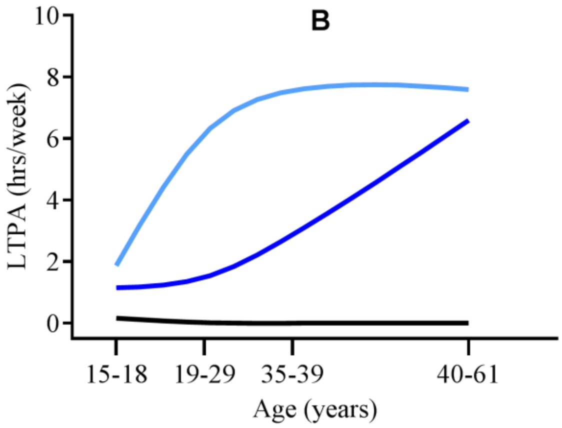

Trajectory	Men	Women
3	0.72 (0.68, 0.77)	0.66 (0.61, 0.70)
2	0.67 (0.63, 0.70)	0.62 (0.58, 0.66)
1	1.00 (Referent)	1.00 (Referent)

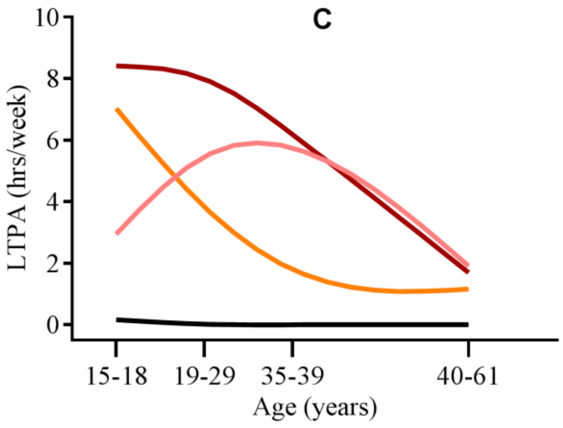

Trajectory	Men	Women
6	0.95 (0.90, 0.99)	1.00 (0.94, 1.06)
4	0.95 (0.90, 1.01)	0.88 (0.82, 0.94)
9	0.87 (0.83, 0.91)	0.86 (0.80, 0.91)
1	1.00 (Referent)	1.00 (Referent)

eFigure 3. Leisure-Time Physical Activity (LTPA) Trajectories and Respective Hazard Ratios (95% Confidence Intervals) for Men (n=183,453) and Women (n=131,606). Hazard ratios are provided for all-cause mortality among maintainers (A), increasers (B), and decreasers (C). Hazard ratios

© 2019 Saint-Maurice PF et al. *JAMA Network Open*.

were computed separately for all-cause mortality outcome using trajectory 1 as the referent group. Hazard ratios were adjusted for: age, sex, education, race, smoking status/dose, total energy intake, diet percent fat, alcohol consumption, red meat consumption, consumption of fruits, consumption of vegetables, vitamin/mineral supplementation and, body mass index at age 18 years.

© 2019 Saint-Maurice PF et al. *JAMA Network Open*.

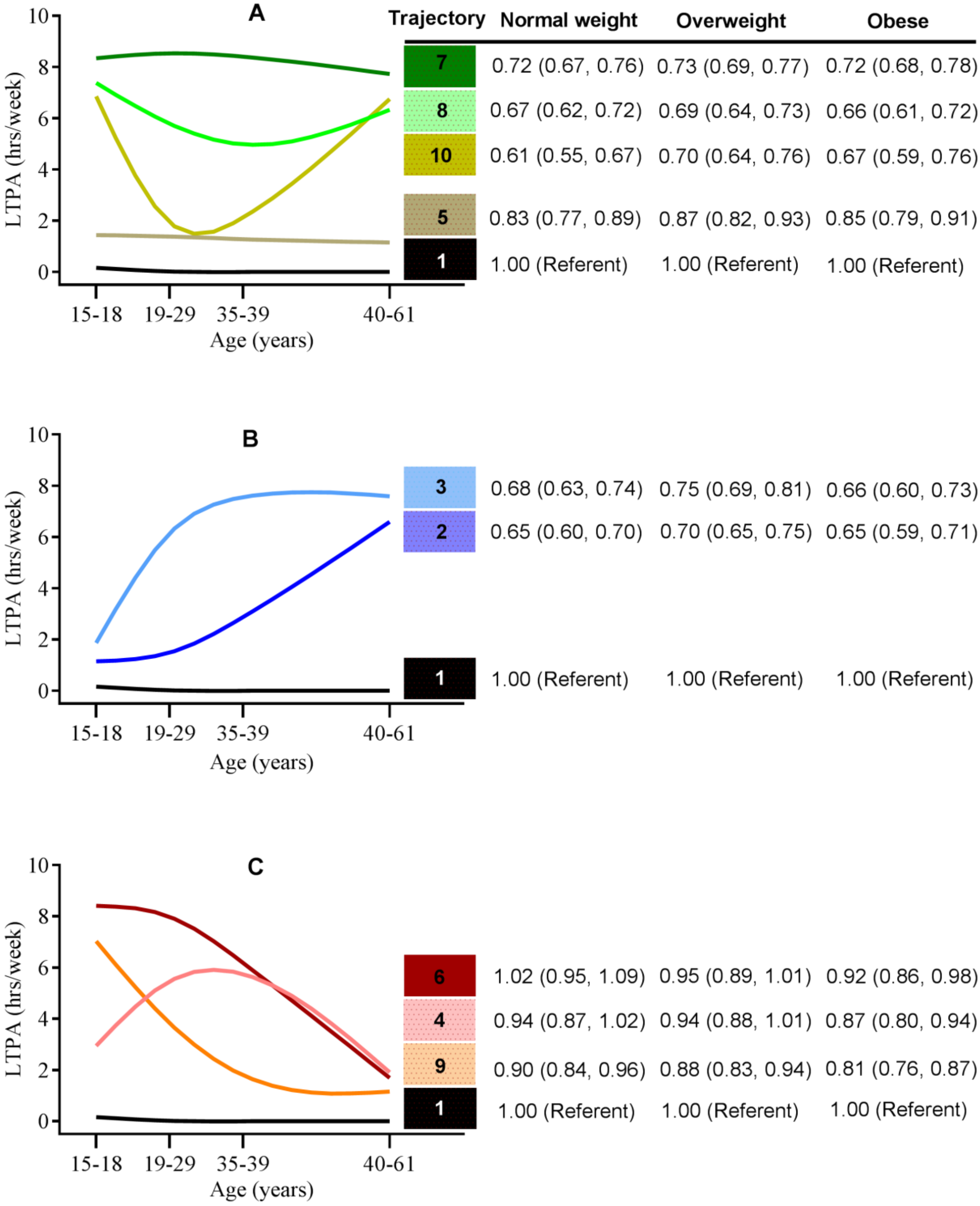

	Hazard ratios (95% CI)		
Trajectory	Normal weight	Overweight	Obese
**A**			
7	0.72 (0.67, 0.76)	0.73 (0.69, 0.77)	0.72 (0.68, 0.78)
8	0.67 (0.62, 0.72)	0.69 (0.64, 0.73)	0.66 (0.61, 0.72)
10	0.61 (0.55, 0.67)	0.70 (0.64, 0.76)	0.67 (0.59, 0.76)
5	0.83 (0.77, 0.89)	0.87 (0.82, 0.93)	0.85 (0.79, 0.91)
1	1.00 (Referent)	1.00 (Referent)	1.00 (Referent)
**B**			
3	0.68 (0.63, 0.74)	0.75 (0.69, 0.81)	0.66 (0.60, 0.73)
2	0.65 (0.60, 0.70)	0.70 (0.65, 0.75)	0.65 (0.59, 0.71)
1	1.00 (Referent)	1.00 (Referent)	1.00 (Referent)
**C**			
6	1.02 (0.95, 1.09)	0.95 (0.89, 1.01)	0.92 (0.86, 0.98)
4	0.94 (0.87, 1.02)	0.94 (0.88, 1.01)	0.87 (0.80, 0.94)
9	0.90 (0.84, 0.96)	0.88 (0.83, 0.94)	0.81 (0.76, 0.87)
1	1.00 (Referent)	1.00 (Referent)	1.00 (Referent)

eFigure 4. Leisure-Time Physical Activity (LTPA) Trajectories and Respective Hazard Ratios (95% Confidence Intervals) for All-Cause Mortality Among Maintainers (A), Increasers (B), and Decreasers (C). Hazard ratios are adjusted for: age, sex, education, race, smoking status/dose,

© 2019 Saint-Maurice PF et al. *JAMA Network Open*.

total energy intake, diet percent fat, alcohol consumption, red meat consumption, consumption of fruits, consumption of vegetables, vitamin/mineral supplementation and, body mass index at age 18 years. Hazard ratios are stratified for BMI classification [normal weight (n=114,450), overweight (n=129,631), and obese (n=64,917)] at 50-71 years (i.e., baseline) using participants in trajectory 1 as the referent group. Body mass index at 50-71 years from 6,061 participants were missing.

© 2019 Saint-Maurice PF et al. *JAMA Network Open*.
